# Dogs with sepsis are more hypercoagulable and have higher fibrinolysis inhibitor activities than dogs with non-septic systemic inflammation

**DOI:** 10.3389/fvets.2025.1559994

**Published:** 2025-04-30

**Authors:** Emily Hill, Yao Zhu, Marjory B. Brooks, Robert Goggs

**Affiliations:** ^1^Department of Clinical Sciences, College of Veterinary Medicine, Cornell University, Ithaca, NY, United States; ^2^Comparative Coagulation Laboratory, Department of Population Medicine and Diagnostic Sciences, College of Veterinary Medicine, Cornell University, Ithaca, NY, United States

**Keywords:** sepsis, dogs, fibrinolysis, antithrombin, antiplasmin, TAFI, PAI-1, nucleosomes

## Abstract

**Introduction:**

Hemostatic imbalance in dogs with sepsis is characterized by hypercoagulability and hypofibrinolysis. We aimed to determine whether these abnormalities are unique features of sepsis or are also present in dogs with non-septic critical illness. Secondary aims were to assess relationships between coagulation assay results and circulating markers of neutrophil extracellular traps (NETs), and to relate coagulation assay abnormalities with survival in dogs with sepsis.

**Methods:**

This prospective single-center observational cohort study enrolled 55 client-owned dogs that satisfied at least 2 systemic inflammatory response syndrome (SIRS) criteria. Dogs with a bacterial infection were categorized as sepsis, those without evidence of infection were categorized as non-infectious systemic inflammation (nSIRS). Clotting times, fibrinogen and D-dimer concentrations, and activities of antithrombin (AT), antiplasmin (AP), thrombin activatable fibrinolysis inhibitor (TAFI), and total and active plasminogen activator inhibitor-1 (PAI-1) were measured. Thrombin generation and overall hemostasis potential assays were performed and concentrations of cell-free DNA (cfDNA) and H3.1 nucleosomes quantitated.

**Results:**

Compared to dogs with nSIRS, dogs with sepsis had higher fibrinogen concentrations, greater endogenous thrombin potential, higher AP and TAFI activities and greater overall hemostasis and coagulation potential values. H3.1 nucleosome and cfDNA concentrations were strongly correlated and significantly associated with various coagulation variables. In dogs with sepsis, non-survivors had lower AT activity, and higher active PAI-1 and H3.1 nucleosome concentrations.

**Discussion:**

Relative to non-septic critically ill dogs, dogs with sepsis are hyperfibrinogenemic, hypercoagulable and have higher AP and TAFI activities. Concentrations of H3.1 nucleosomes and active PAI-1 and AT activity might have prognostic value in dogs with sepsis.

## Introduction

Sepsis is a major cause of morbidity and mortality in dogs (1), and is defined in humans as the dysregulated host response to infection that causes organ dysfunction (2) and loss of hemostatic balance (3). Sepsis is associated with development of a thromboinflammatory state (4, 5) that can manifest as thrombosis (6–8) and consumptive coagulopathy associated with disseminated intravascular coagulation (9). The hypercoagulable state in sepsis results from proinflammatory cytokine activity (10–12), intravascular tissue factor expression (13), and diminished concentrations of endogenous inhibitors and anticoagulants, including antithrombin (AT) and protein C (3, 5). In humans, fibrinolytic pathway dysfunction contributes to the coagulopathy in sepsis (14), and is associated with organ dysfunction and mortality (15). Plasmin, the primary fibrinolytic enzyme, is generated through plasminogen cleavage by activating enzymes including tissue plasminogen activator (tPA) and urokinase. Increased tPA activity in sepsis promotes fibrinolysis (16), but is opposed by simultaneous increases in fibrinolysis inhibitors (17). Plasminogen activator inhibitor-1 (PAI-1), the primary inhibitor of tPA, is upregulated in humans with sepsis (17, 18), and dogs with immune-mediated hemolytic anemia (IMHA) (19), but data on the role of PAI-1 in dogs with sepsis are limited (20). Fibrinolysis is also inhibited by the plasma carboxypeptidase, thrombin activatable fibrinolysis inhibitor (TAFI) and the serine protease inhibitor antiplasmin (AP). In humans with sepsis, decreased TAFI activity is associated with organ dysfunction (21), and death (22). Dogs with sepsis have lower AT and higher AP and TAFI activity compared to healthy controls, and this higher TAFI activity is strongly correlated with reduced overall fibrinolysis potential in a microtiter plate fibrinolysis assay (23).

The innate immune response to infection contributes to organ dysfunction in sepsis through the formation of neutrophil extracellular traps (NETs) and the process of immunothrombosis (24). Immunothrombosis may be protective when localized but contributes to morbidity and mortality when excessive or dysregulated (25). Accumulating evidence indicates that NET formation (NETosis) occurs in dogs (24, 26) and might contribute to sepsis pathogenesis. These structures consist of extracellular DNA decorated with bactericidal cellular proteins, including elastase, myeloperoxidase, and cathepsins (27), and can be identified by measurements of plasma cell-free DNA (cfDNA), nucleosomes, and high-mobility group box-1 (HMGB-1) (28–30). Plasma cfDNA concentrations are significantly higher in dogs with bacteremia compared to those with negative blood cultures, suggesting that circulating cfDNA might derive from intravascular NETosis (29). The structural components of NETs can directly activate platelets, facilitate thrombus formation, and inhibit fibrinolysis and the natural anticoagulant pathways (24, 27). In dogs, NET components accelerate clot formation, while neutrophil-derived cfDNA decreases fibrinolysis (31), by impairing plasmin’s access to fibrin (32).

Coagulation disturbances and fibrinolytic disorders are well-recognized in sepsis, but occur in other types of critical illness in humans, including liver failure (33), trauma (34), burns (35), heatstroke (36), cancer (37), and acute respiratory failure (38). As such, it is unknown if the hypofibrinolysis documented in dogs with sepsis is specific to infection or is a feature of critical illness irrespective of cause. We addressed this question by comparing cohorts of dogs with sepsis and non-septic critical illness. Secondary aims were to assess the relationships between NET components and coagulation assay results, and to evaluate the association between coagulation disturbances and survival in dogs with sepsis. We hypothesized that compared to dogs with non-septic critical illness, dogs with bacterial sepsis are hypofibrinolytic and have higher PAI-1 activity. Additional hypotheses were that in dogs with sepsis, increased NETosis biomarkers correlate with hypofibrinolysis, and that non-survivors are hypofibrinolytic relative to survivors.

## Materials and methods

### A priori sample size calculations

To estimate the necessary sample size for this study, TAFI activity data from a previous study of dogs with sepsis were used (23). In that study, the mean ± SD TAFI activity was 63% ± 27 in dogs with sepsis compared to 46% ± 21 in healthy controls. If dogs with sepsis and non-septic critical illness had comparable TAFI activities to those previously observed, 26 dogs per group would be required to detect an equivalent difference with 80% power at *p* < 0.05.

### Study design

This was a prospective observational study at an academic teaching hospital conducted over a 12-month period from 07/2023 to 08/2024. Client-owned dogs weighing >10 kg presented to the Cornell University Hospital for Animals were eligible for enrollment if they had evidence of a severe systemic illness or injury and met ≥2 criteria of the systemic inflammatory response syndrome (SIRS): temperature (<37.8°C or >39.4°C; <100.2°F or >103.1°F); heart rate >140 bpm; respiratory rate >20 bpm, leukocyte count <6 × 10^3^/μL or >16 × 10^3^/μL or 3% band neutrophils (3, 39). Scoring for SIRS criteria was performed at rest in the emergency room or ICU. When dogs were persistently panting, respiratory rate was excluded as a SIRS criterion. Bacterial sepsis was defined as the presence of SIRS with a documented or highly suspected infection (40, 41). Dogs were excluded if a fungal, parasitic, or viral cause of sepsis was documented or suspected; if a neoplastic disease process was present; or if immunosuppressive or antithrombotic treatments had been administered in the preceding 14 days. Dogs with severe anemia (packed cell volume <20%) were also excluded. All dogs were enrolled with documented informed owner consent. If owners were absent at the time of study enrollment, they were contacted by telephone, consent was obtained and the study documents shared electronically immediately after the telephone conversations. The study protocol was approved by the Institutional Animal Care and Use Committee (Cornell IACUC Protocol #2014-0053).

### Patient data collection

Data recorded at the time of study enrollment included signalment, body weight (kg), heart rate (bpm), respiratory rate (bpm), temperature (°F), blood pressure (mmHg), physical examination findings including modified Glasgow coma scale score (3–18), blood oxygen saturation by pulse oximetry (SpO_2_, %), mentation status (0–4) and body cavity fluid score (0, 1 or 2). Subsequently, once the required clinicopathologic data were available, rapid and complete illness severity scores were calculated (acute patient physiologic and laboratory evaluation; APPLE_fast_, APPLE_full_), with missing data handled as recommended (42). Final diagnosis was recorded and outcome status classified based on survival to hospital discharge, or death or euthanasia for disease severity or deterioration of condition. After establishing a final diagnosis and compiling test results, dogs were categorized into two groups, bacterial sepsis or non-infectious systemic inflammation (nSIRS). Where available, diagnostic imaging reports, necropsy findings, cavitary effusion cytology results, fine needle aspirate biopsy findings, and skin scraping cytology reports were reviewed to confirm correct group assignment.

### Blood sample collection

Blood samples were collected upon initial intravenous catheter placement whenever possible, or by direct venipuncture when necessary. Point-of-care venous blood gases, electrolytes, hematocrit, and lactate concentrations were analyzed immediately in heparinized whole blood (RapidPoint 500, Siemens Healthcare, Malvern, PA). In the following order, blood was collected directly into evacuated tubes (Vacutainer, BD and Co., Franklin Lakes, NJ) containing no-additive for serum biochemistry, 3.2% sodium citrate (1:9 ratio) for coagulation testing, and K_2_-EDTA for complete blood counts (CBC) and plasma H3.1 nucleosome concentration measurements. Automated CBCs (ADVIA 2120, Siemens Healthcare) with blood smear review by board-certified veterinary clinical pathologists and serum biochemistry profiles (Cobas C501, Roche Diagnostics, Indianapolis, IN) were performed at the Animal Health Diagnostic Center (AHDC, Cornell University, Ithaca, NY) as soon as possible and always within 72 h of sample collection. Where necessary, serum samples were refrigerated (4°C) or frozen (−20°C) pending biochemistry analyses and EDTA samples refrigerated pending analysis. Citrate and EDTA whole blood samples were centrifuged for 10 min at 1,370 g (Ultra-8 V Centrifuge, LW Scientific, Lawrenceville, GA). To minimize freeze–thaw cycling during subsequent analysis, citrate and EDTA plasma samples were aliquoted into 1.5 mL freezer tubes (Polypropylene Screw-Cap Microcentrifuge Tubes, VWR, Radnor, PA) with particular care taken to avoid disruption of the cell pellet and frozen immediately at −80°C until batch analysis.

### Coagulation and fibrinolysis analyses

All hemostasis testing was performed at an American Association of Veterinary Diagnostic Laboratories accredited reference laboratory (Comparative Coagulation Laboratory, AHDC). Coagulation profiles, consisting of activated partial thromboplastin time (APTT), prothrombin time (PT), clottable (Clauss) fibrinogen, antithrombin activity (AT), and D dimer concentration were performed as previously described in detail (9). All assays were performed on an automated coagulation instrument (STACompact Max, Diagnostica Stago, Parsippany NJ) using commercial coagulation reagents and kits (Thromboplastin LI, Helena Diagnostics, Beaumont, TX; Actin FS, Siemens Healthcare; Stachrom AT III and STA Fibrinogen, Diagnostica Stago; and HemosIL D-dimer, Werfen, Bedford, MA). Fibrinogen and AT assays were modified by use of a canine plasma standard. D dimer was assayed with the manufacturer’s human standard and results reported as ng/mL D dimer units (DDU). Antiplasmin activity (AP) and plasminogen activity were measured in chromogenic assays as previously described (23). In brief, AP was measured based on inhibition of a human plasmin reagent using a commercially available human AP kit (Plasmin Inhibitor, HemosIL, Werfen) Plasminogen activity was measured in acidified and neutralized plasma activated by urokinase (Prospec, East Brunswick, NJ), prior to reaction with a plasmin substrate (S-2251, Diapharma, West Chester, OH). Plasminogen activities were reported relative to a pooled dog plasma standard assigned a value of 100%.

Quantitation of TAFI activity was performed using a chromogenic assay kit (Pefakit TAFI, dsm-firmenich, Aesch, Switzerland), as previously described (23). Samples were assayed in duplicate, with mean activities of replicates reported as the percentage activity of a pooled human plasma TAFI calibrator.

Fibrin clot formation and lysis were evaluated in the overall hemostasis potential assay (OHP) (43), a kinetic turbidimetric assay, modified for canine plasma as previously described (44, 45). Coagulation reactions were activated with bovine alpha-thrombin (final concentration 0.05 U/mL), with paired lysis reactions triggered by the addition of human recombinant tPA (final concentration 350 ng/mL). Three parameters were derived: overall coagulation potential (OCP), defined as the area under the coagulation curve; OHP, defined as the area under the lysis curve; and overall fibrinolysis potential (OFP), calculated as the relative difference between OCP and OHP values: OFP % = (OCP – OHP)/OCP × 100.

Thrombin generation (TG) was measured by the calibrated automated thrombogram method using a dedicated spectrofluorimeter and software (Thrombinoscope, Diagnostica Stago), as previously described (46). Plasma samples were diluted 1:2 in imidazole buffered saline and activated with a thromboplastin reagent containing 1 pM tissue factor (PPP-reagent low, Diagnostica Stago). Assays were performed in duplicate with the mean values compiled for the following parameters: lag time (min), peak thrombin (nM), time to peak (min) and overall endogenous thrombin potential (ETP, nM·min).

### NET biomarkers

H3.1 nucleosome concentrations in EDTA plasma were measured in duplicate using a validated commercial ELISA kit and manufacturer provided standards and controls (Volition Nu.Q Discover H3.1, DiaPharma) (47–50). Pooled healthy dog plasma was included as an additional control. Insufficient EDTA plasma was available for one nSIRS dog; citrate plasma was used instead. Analyses were repeated for any samples with an intra-assay coefficient of variation (CV) > 10% and mean values used for further analyses. Samples with measured concentrations above the standard curve were diluted 1:4 or 1:10 with assay buffer and re-analyzed. Where insufficient sample volume precluded repeated analysis, the highest possible concentration for the relevant standard curve corrected for 1:4 or 1:10 dilution was recorded and the whole H3.1 nucleosome dataset assumed to be non-parametric. Samples and reagents were homogenized at room temperature prior to microplate loading, incubated at room temperature for 150 min on an orbital shaker (ThermoMixer FP, Eppendorf, Enfield, CT) then washed 3 times using an automated plate washer (50TS Microplate Washer, BioTek Agilent). H3.1 nucleosomes were detected using a horseradish peroxidase-conjugated detection antibody incubated for 90 min at room temperature with shaking. After plate washing, colorimetric substrate solution was added, the plate incubated in the dark until color development was sufficient, and reactions stopped by addition of an acid solution. Light absorbance at 450 nm was measured using a microplate reader (Cytation 1, BioTek Agilent) and unknown concentrations derived from a curve plotted using manufacturer supplied standards fitted with a 4-parameter model (Gen5, BioTek Agilent). Concentrations of cfDNA were measured in citrate plasma samples by fluorimetry (Qubit 4.0, Invitrogen, Carlsbad, CA) as previously described (51). Manufacturer’s instructions and standards were used for assay calibration (Quant-iT hsDNA assay kit; Invitrogen). Test plasma samples were run in duplicate and mean values used for further analyses. Where the intra-assay CV was >10%, additional analyses were performed to provide two consistent readings.

### Plasminogen activator inhibitor-1

Concentrations of total and active PAI-1 in citrate plasma samples were determined using commercial canine-specific ELISA kits (Molecular Innovations, Novi, MI) as previously reported (20). The total PAI-1 assay is a conventional sandwich ELISA assay employing proprietary capture and detection antibodies raised against canine PAI-1 and measures all PAI-1 forms (free, latent, complexed) present in the test samples. The active PAI-1 assay is based on binding of functionally active plasma PAl-1 to a urokinase-coated microtiter plate. Latent or complexed PAl-1 does not bind urokinase and is washed away prior to addition of an anti-PAl-1 antibody. After a subsequent wash step, bound primary antibody is detected using a secondary antibody conjugated to horseradish peroxidase. A colorimetric substrate is then added such that the color generated is directly proportional to the concentration of active PAl-1 in the sample. The requisite (but not supplied) buffers were made according to the kit manufacturer’s specifications using standard laboratory reagents (Sigma-Aldrich, St. Louis, MO; ThermoFisher Scientific, Waltham, MA). Assays were conducted according to the kit manufacturer’s instructions with all 30 min incubation steps occurring at room temperature with orbital shaking (ThermoMixer FP, Eppendorf). Automated plate washing and colorimetric detection at 450 nm was performed as described above for H3.1 nucleosome quantitation.

### Statistical analyses

Statistical analyses were performed using commercial software (Prism 10.4.1, GraphPad, Boston, MA). Descriptive statistics were produced for each group of dogs after assessing data for normality using the D’Agostino Pearson test. Group comparisons of continuous data were evaluated via unpaired *t*-tests with Welch’s correction for parametric data, whereas nonparametric data were compared using the Mann–Whitney U test. Correlations between coagulation parameters were assessed using Spearman’s coefficients and scatterplots. Strength of correlation was defined as follows: <0.5 weak, 0.5–0.6 mild, 0.6–0.7 moderate, 0.7–0.8 strong, 0.8–0.9 very strong, and 0.9–1.0 excellent. Comparisons between groups based on *a priori* hypotheses (e.g., for coagulation variables) were not corrected for multiple comparisons. A Bonferroni correction was applied to comparisons of demographic, clinical and clinicopathologic data between groups (as noted below).

## Results

### Animals

A total of 55 dogs were enrolled, including 28 dogs in the bacterial sepsis group and 27 dogs in the nSIRS group. Dogs with bacterial sepsis had septic peritonitis (*n* = 8), pyometra (*n* = 5), tickborne disease (*n* = 5), aspiration pneumonia (*n* = 3), and bacterial pneumonia (*n* = 2). Other causes of sepsis included mastitis, necrotizing hepatitis, retrobulbar abscess, bite wounds, and otitis interna (all *n* = 1). Dogs in the nSIRS group had gastric dilatation and volvulus syndrome (*n* = 7), vehicular trauma (*n* = 4), severe hepatopathy (*n* = 3), pancreatitis (*n* = 2), porcupine quill impalement (*n* = 2), bite injuries (*n* = 2), and non-perforated intestinal obstruction, liver lobe torsion, acquired portosystemic shunt, uroperitoneum, non-associative immune thrombocytopenia, cluster seizures, and tremorgenic toxin ingestion (all *n* = 1).

Population characteristics, clinical assessment parameters and illness severity scores are summarized in Table 1. After adjustment for multiple comparisons, none of the differences between the two populations were significant. Acid–base, electrolyte and lactate measurements obtained point-of-care at study enrollment and clinicopathologic data derived from admission complete blood count and serum biochemistry profiles are summarized in Supplementary Tables S1–S3. After adjustment for multiple comparisons, 2 differences between the groups were identified. Compared to dogs with sepsis, dogs with nSIRS had significantly lower mean corpuscular hemoglobin concentrations (MCHC, median (IQR) 33.0 g/dL (33.0–34.0) versus 32.5 g/dL (32.0–33.0)) and significantly higher alanine aminotransferase activities (ALT, 152 U/L (62–294) versus 49 U/L (31–54)). Samples of blood, urine, peritoneal effusion, uterus, and lung tissue were submitted for bacterial culture and susceptibility testing from 12 dogs in the bacterial sepsis group with 8 positive results. Isolated bacteria are summarized in Supplementary Table S4. Two negative culture results were returned for 2 nSIRS dogs.

**Table 1:** Summary of population characteristics, clinical assessment parameters and illness severity scores for dogs with sepsis compared to dogs with nSIRS.

Variable (unit, range or limit)	Sepsis (*n* = 28)	nSIRS (*n* = 27)	Unadjusted *p*
Age (Y)	5.3 ± 3.5	6.1 ± 3.4	0.393
Bodyweight (kg)	26.9 ± 8.6	31.8 ± 9.2	0.050*
Sex (F/FS/M/MN)	8/8/4/8	3/11/4/9	–
Heart rate (bpm)	145 ± 22	169 ± 36	0.006*
Respiratory rate (bpm)	36 (29–44)	40 (30–48)	0.479
Temperature (°C)	39.4 (38.9–40.0)	38.9 (37.8–39.4)	–
Temperature (°F)	103 (102–104)	102 (100–103)	0.006*
Systolic pressure (mmHg)	144 ± 23	145 ± 18	0.916
Diastolic pressure (mmHg)	82 (75–91)	92 (69–105)	0.471
Mean arterial pressure (mmHg)	98 ± 20	101 ± 20	0.543
Oxygen saturation (SpO_2_, %)	98 (95–99)	97 (95–99)	0.917
MGCS score (3–18)	18 (17–18)	18 (17–18)	1.00
Mentation score (Max. 4)	1 (0–1)	0 (0–1)	1.00
Fluid score (Max. 2)	0 (0–1)	0 (0–1)	1.00
SIRS criteria (*n* of 4)	3 (3–4)	3 (2–3)	0.016*
APPLE_full_ (Max. 80)	24 (18–29)	22 (13–30)	0.522
APPLE_fast_ (Max. 50)	22 (16–28)	20 (15–25)	0.572

Parametric data are summarized as mean ± standard deviation and compared between groups using unpaired *t*-tests with Welch’s correction. Nonparametric data are summarized as median (interquartile range) and compared between groups by Mann–Whitney U test. None of the differences observed between groups were significantly different after Bonferroni correction for multiple (*n* = 15) comparisons. * Corrected *p*-values: Bodyweight = 0.745; Heart rate = 0.086; Temperature 0.093; SIRS criteria = 0.243. APPLE, acute patient physiologic and laboratory evaluation; bpm, beats or breaths per minute; F, female; FS, female-spayed; M, male; MN, male-neutered; MGCS, modified Glasgow coma scale score; SIRS, systemic inflammatory response syndrome.

### Coagulation, fibrinolysis and NETosis biomarker analyses

**Figure 1:**
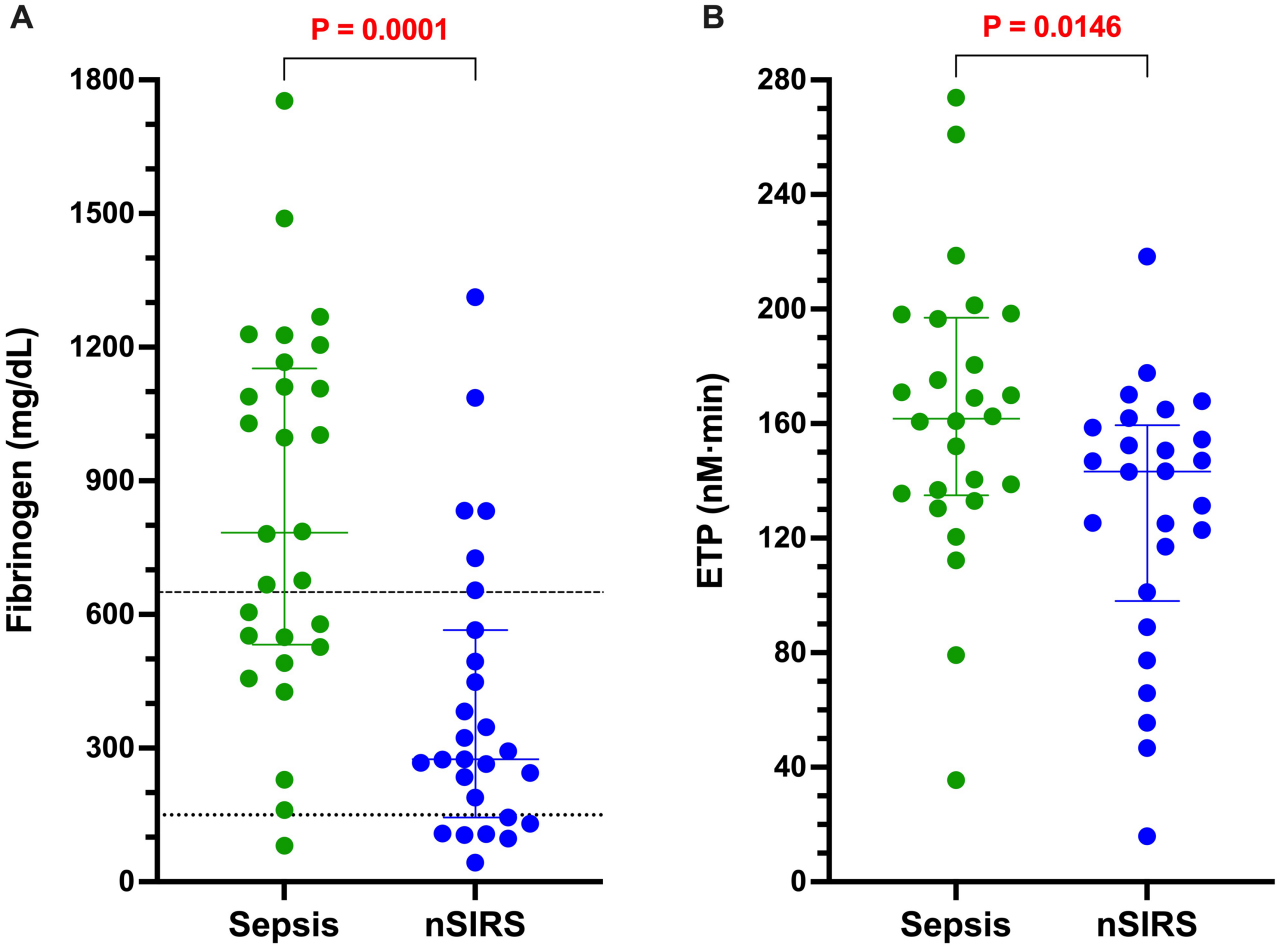
Dogs with bacterial sepsis have significantly greater concentrations of clottable fibrinogen and higher endogenous thrombin potential (ETP) compared to dogs with non-septic systemic inflammation (nSIRS). Dotplots of **(A)** Clauss fibrinogen concentration (mg/dL), and **(B)** endogenous thrombin potential (derived from the area under the thrombin generation curve, nM × min) in dogs with sepsis compared to dogs with nSIRS. Long horizontal lines represent the median value, whiskers represent the interquartile range; data were compared with the Mann–Whitney U test with alpha set at 0.05. Horizontal dotted lines represent the laboratory reference interval for fibrinogen.

**Figure 2:**
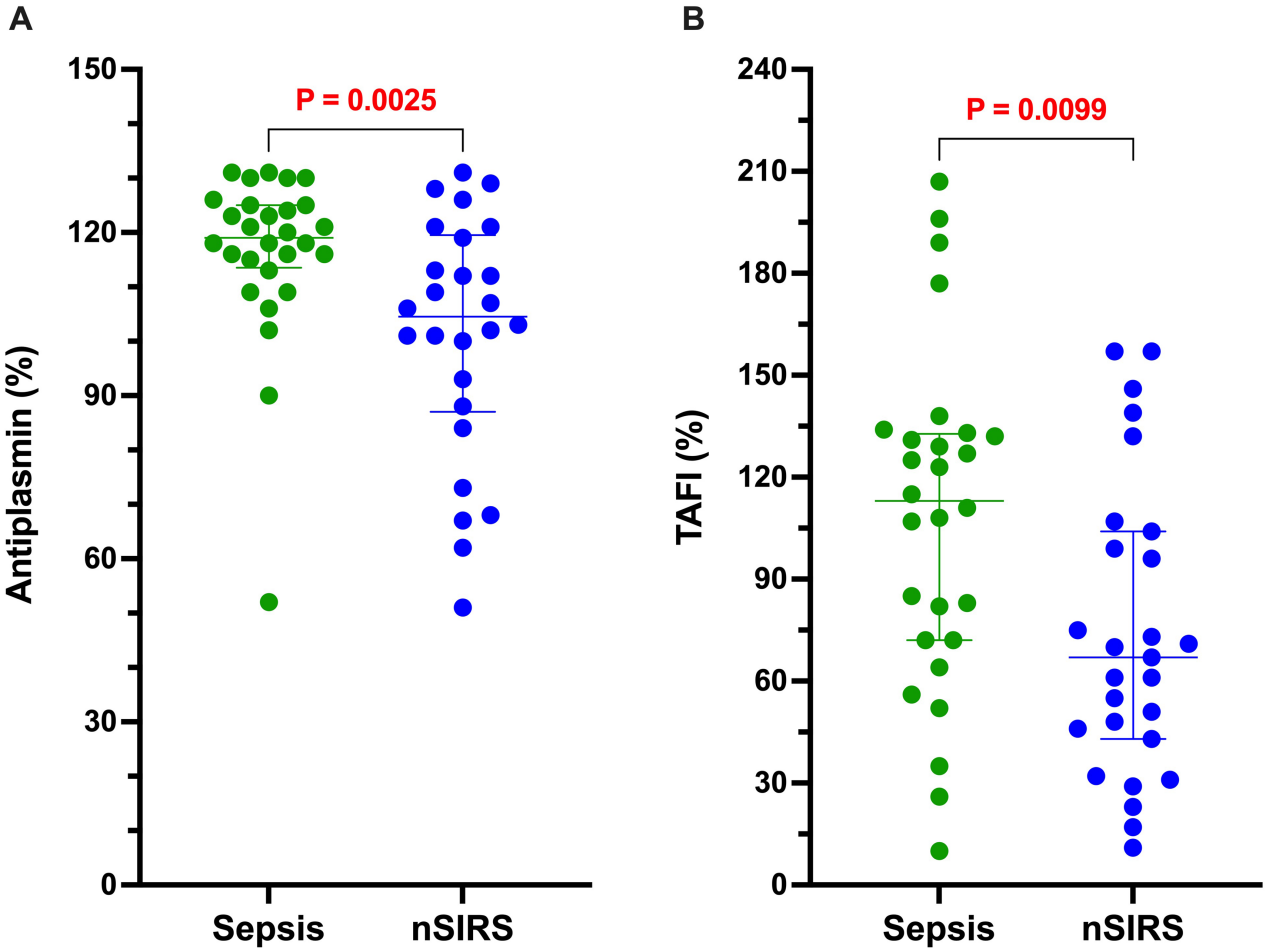
Dogs with sepsis have a significantly greater antiplasmin (AP) and thrombin-activable fibrinolysis inhibitor (TAFI) activities than dogs with non-septic systemic inflammation (nSIRS). Dotplots of **(A)** AP (%), and **(B)** TAFI activity (%). Long horizontal lines represent the median value, whiskers represent the interquartile range; data were compared with the Mann–Whitney U test with alpha set at 0.05.

**Figure 3:**
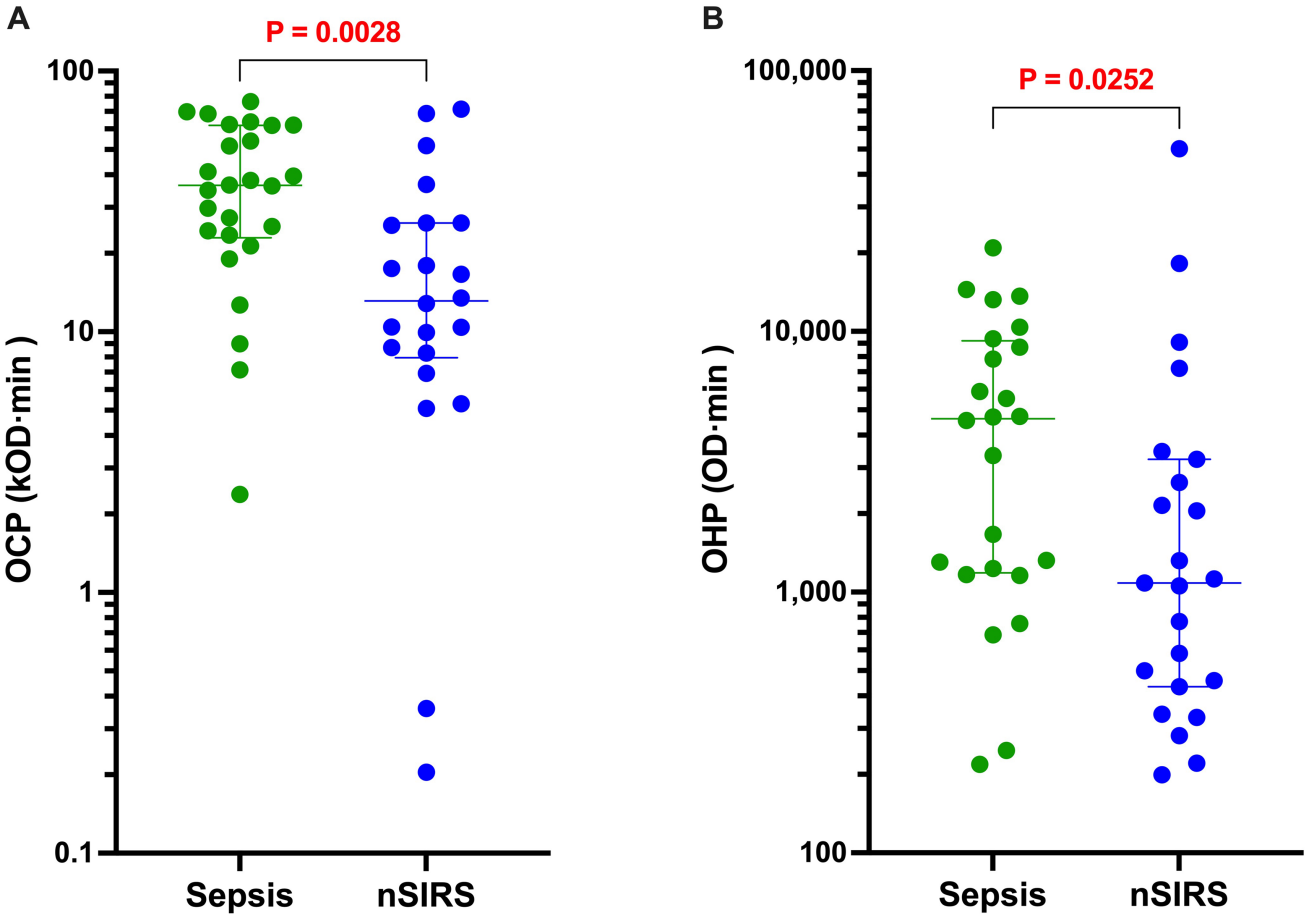
Dogs with sepsis have significantly greater overall coagulation potential (OCP) and overall hemostatic potential (OHP) than dogs with non-septic systemic inflammation (nSIRS). Dotplots of **(A)** OCP (OD value ×10^3^ × min), and **(B)** OHP (OD value × min) of dogs with sepsis compared to dogs with nSIRS. Long horizontal lines represent the median value, whiskers represent the interquartile range; data were compared with the Mann–Whitney U test with alpha set at 0.05.

The results of all coagulation and fibrinolysis testing and the concentrations of biomarkers of NET formation are summarized in Table 2. Relative to dogs with nSIRS, dogs with bacterial sepsis had significantly higher fibrinogen concentrations (Figure 1A), greater ETP values (Figure 1B), higher AP and TAFI activities (Figure 2), and greater OCP and OHP values (Figure 3). No differences between the two groups were observed for the other coagulation parameters, the other derived thrombin generation variables, the concentrations of plasminogen or PAI-1 or the OFP (Supplementary Figures S1–S3). Similarly, no differences were observed between the plasma concentrations of cfDNA or H3.1 nucleosomes in the two groups (Supplementary Figure S4).

**Table 2:** Summary of coagulation, fibrinolysis and NET formation biomarkers data for dogs with sepsis compared to dogs with nSIRS.

Variable	Sepsis (*n* = 28)	nSIRS (*n* = 27)	*p*
Coagulation parameters			
Activated partial thromboplastin time (s)	14.8 (12.6–16.4)	13.4 (12.5–17.9)	0.877
Prothrombin time (s)	12.9 (12.3–13.9)	12.7 (12.3–14.1)	0.805
Antithrombin activity (%)	84.9 ± 22.5	84.0 ± 31.9	0.905
Clottable fibrinogen (Clauss) (mg/dL)	784 (533–1,152)	275 (144–565)	**0.000**
D-dimer (ng/mL)	771 (330–1864)	663 (417–1,598)	0.924
Thrombin generation			
Lag time (min)	3.0 (2.7–3.9)	2.7 (2.3–3.3)	0.080
Time to peak (min)	6.2 (5.2–7.2)	5.4 (5.0–6.5)	0.173
Peak thrombin (nM)	28.4 (19.6–34.2)	26.4 (20.0–31.6)	0.411
Endogenous thrombin potential (nM·min)	162 (135–197)	143 (98–160)	**0.015**
Fibrinolysis parameters			
Plasminogen (%)	105 (68–128)	98 (53–140)	0.898
Antiplasmin activity (%)	119 (114–125)	105 (87–120)	**0.003**
Total plasminogen activator inhibitor-1 (ng/mL)	39.0 (24.5–76.5)	44.0 (21.0–89.0)	0.937
Active plasminogen activator inhibitor-1 (ng/mL)	8.0 (0.9–11.9)	4.9 (0.0–14.5)	0.340
Thrombin-activatable fibrinolysis inhibitor (%)	113 (72–133)	67 (43–104)	**0.001**
Overall hemostatic potential (OHP) assay			
Overall coagulation potential (kOD·min)	36.42 (22.93–61.94)	10.41 (3.90–25.71)	**0.003**
Overall hemostatic potential (OD·min)	3,947 (1058–8,872)	914 (319–2,785)	**0.025**
^1^ Overall fibrinolysis potential (%)	92.4 (75.2–97.3)	89.9 (62.6–94.7)	0.517
NET formation biomarkers			
Cell-free DNA (ng/mL)	500 (420–610)	451 (371–628)	0.710
H3.1 Nucleosomes (ng/mL)	300 (107–642)	164 (35–448)	0.346

Parametric data are summarized as mean ± standard deviation and compared between groups using unpaired *t*-tests with Welch’s correction. Nonparametric data are summarized as median (interquartile range) and compared between groups by Mann–Whitney U test. Corrections for multiple comparisons were not applied because these analyses were conducted based on a priori hypotheses. *P*-values in bold type are considered significant at <0.05 level.

1 Overall fibrinolysis potential (OFP) % = (OCP−OHP/OCP) * 100.

### Correlations in dogs with sepsis

**Figure 4:**
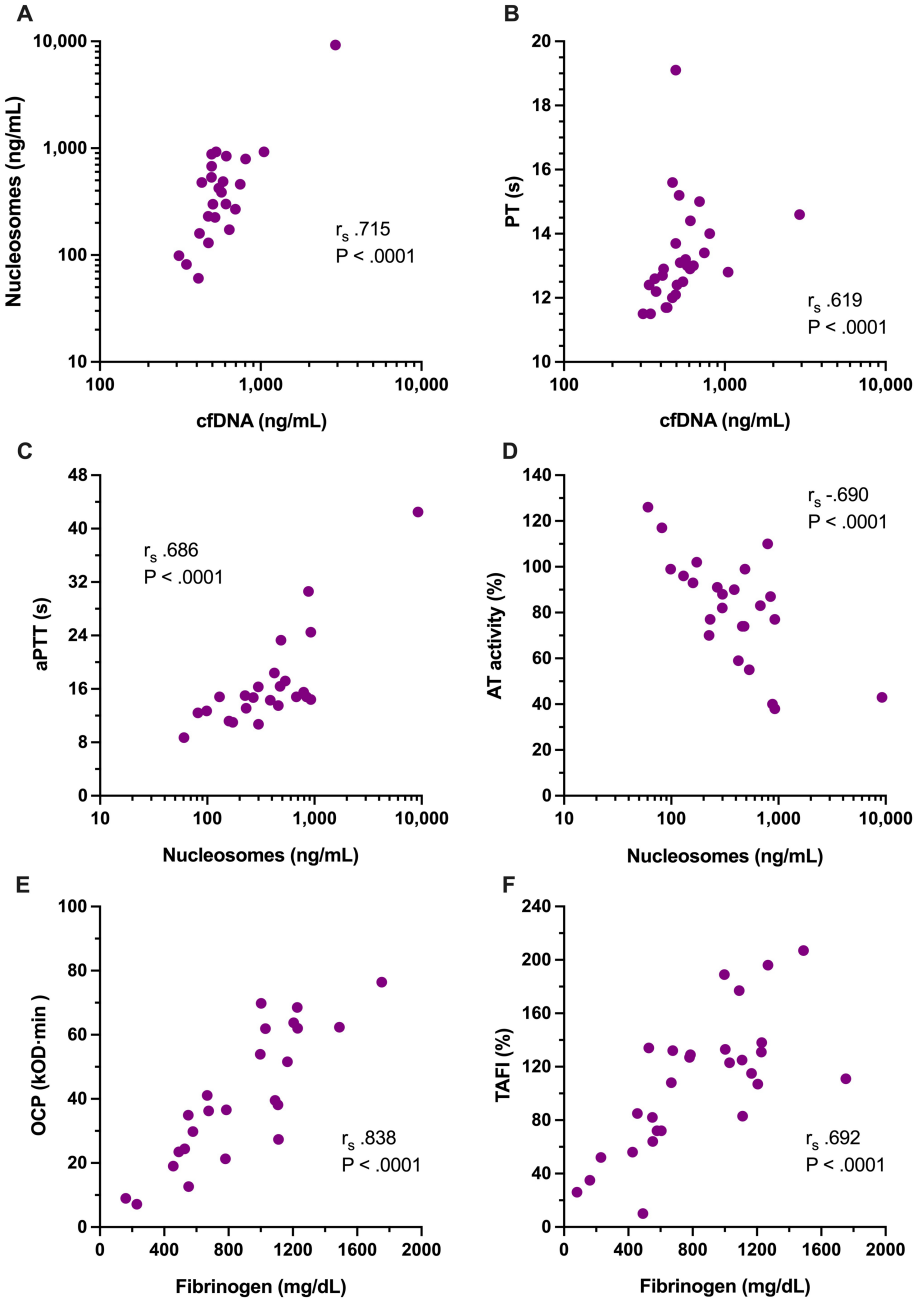
Scatterplots of the significant relationships among coagulation variables and NETosis biomarkers with moderate to very strong Spearman’s correlation coefficients (r_s_), including between **(A)** cell-free DNA (cfDNA, ng/mL) and H3.1 nucleosomes (ng/mL); **(B)** cfDNA and prothrombin time (PT, s); **(C)** H3.1 nucleosomes and activated partial thromboplastin time (aPTT, s); **(D)** H3.1 nucleosomes and antithrombin activity (AT, %); **(E)** overall coagulation potential (OCP, OD value ×10^3^ × min) with clottable fibrinogen concentration; and **(F)** fibrinogen concentration (mg/dL) with thrombin activatable fibrinolysis inhibitor activity (TAFI, %). Relevant correlation coefficients and associated *p*-values are displayed on each panel.

To evaluate the relationships between coagulation, fibrinolysis and NETosis in dogs with sepsis, Spearman’s correlation coefficients were calculated for concentrations of cfDNA and H3.1 nucleosomes against coagulation and fibrinolysis parameters. Concentrations of cfDNA and H3.1 nucleosomes were themselves strongly correlated (r_s_ 0.715, *p* < 0.0001, Figure 4A). Concentrations of cfDNA were moderately correlated with PT (r_s_ 0.619, *p* < 0.0001, Figure 4B) and mildly correlated with lag time (r_s_ 0.535, *p* < 0.0001). H3.1 nucleosome concentrations were moderately correlated with aPTT (r_s_ 0.686, *p* < 0.0001, Figure 4C), mildly correlated with D-dimers (r_s_ 0.584, *p* = 0.001) and moderately, inversely correlated with AT activity (r_s_ − 0.690, *p* < 0.0001, Figure 4D). Neither of the NETosis biomarkers was significantly correlated with OFP.

To assess inter-relationships between the variables that were observed to be different between dogs with sepsis and dogs with nSIRS, Spearman correlation coefficients were calculated between fibrinogen, ETP, AP, TAFI, OCP and OHP. Among these variables, OHP was moderately correlated with ETP (r_s_ 0.624, *p* = 0.001), while OCP was very strongly correlated with fibrinogen (r_s_ 0.838, *p* < 0.0001, Figure 4E), and mildly correlated with TAFI (r_s_ 0.585, *p* = 0.003) and ETP (r_s_ 0.578, *p* = 0.004). Fibrinogen concentration was moderately correlated with TAFI (r_s_ 0.692, *p* < 0.0001, Figure 4F) and mildly correlated with ETP (r_s_ 0.562, *p* = 0.003). Non-parametric correlation coefficients were also calculated to assess the relationships between the OFP (as an overall assessment of fibrinolysis) and the activities of the fibrinolysis inhibitors AP, PAI-1 and TAFI; no significant associations were observed.

### Outcome analyses

**Figure 5:**
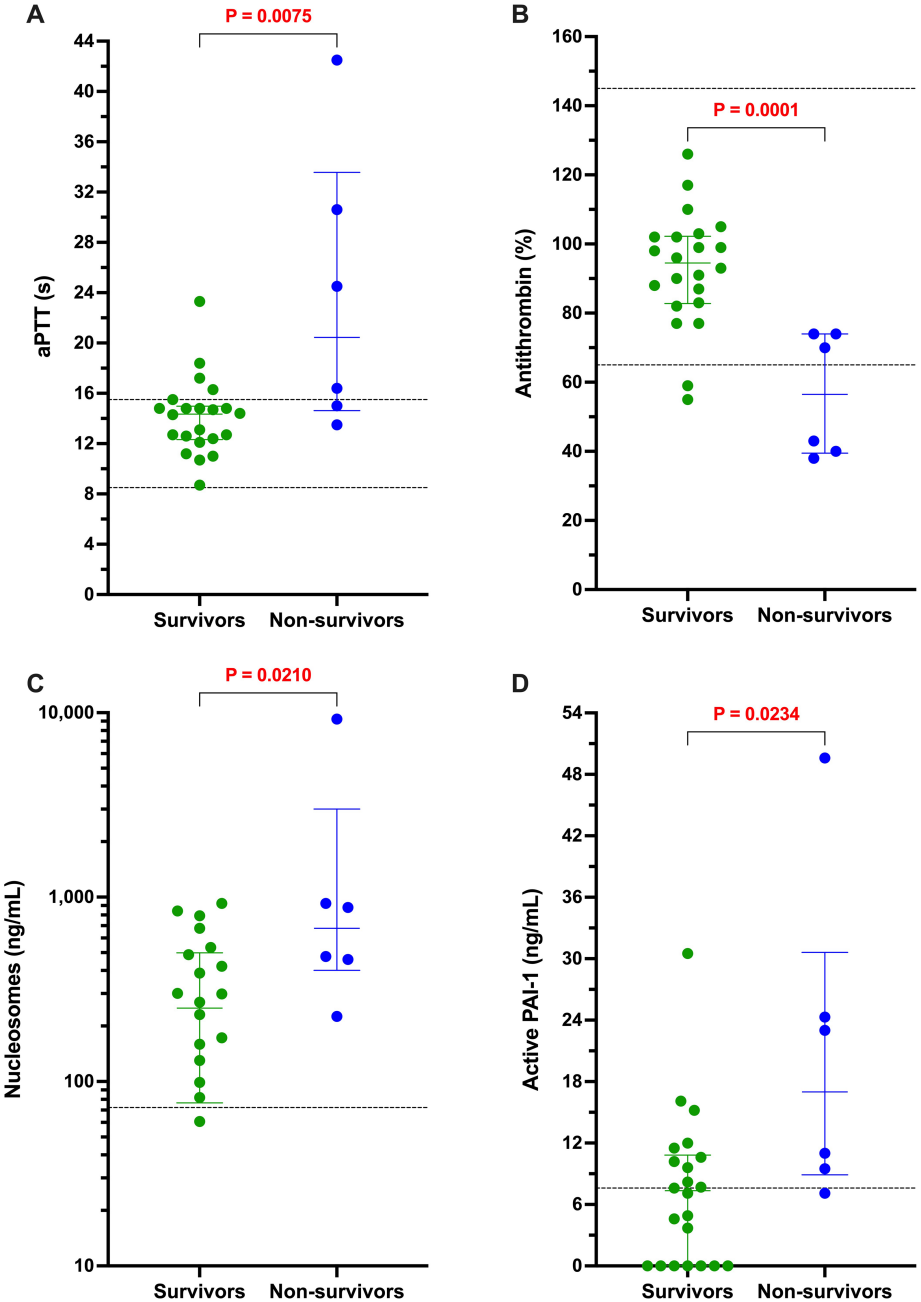
Among dogs with sepsis, non-survivors had longer activated partial thromboplastin times, lower antithrombin activity, and higher concentrations of active PAI-1 and H3.1 nucleosomes compared to survivors. Dotplots of **(A)** activated partial thromboplastin time (aPTT, s), **(B)** antithrombin activity (AT, %), **(C)** active PAI-1 concentration (ng/mL), and **(D)** H3.1 nucleosome concentration (ng/mL) in dogs with sepsis that survived to hospital discharge compared to those that did not. All non-survivors were euthanized due to deterioration in condition or grave prognosis. Data were compared with the Mann–Whitney U test, with alpha set at 0.05. Paired horizontal dotted lines represent the laboratory reference intervals for aPTT and AT. The horizontal dotted line on the PAI-1 panel represents the assay manufacturer supplied normal value for healthy beagle dogs. The horizontal dotted line in panel D represents the mean value for the concentration of H3.1 nucleosomes in the pooled plasma sample derived from 20 healthy dogs.

The case fatality rate at hospital discharge for the whole population was 25.4% (14/55). All non-survivors were euthanized due to deterioration in condition or grave prognosis. Of the dogs with sepsis, 22/28 (78.6%) survived to discharge, whereas 19/27 (70.4%) dogs with nSIRS survived to discharge (*p* = 0.547). In dogs with sepsis, non-survivors had longer activated partial thromboplastin times, lower antithrombin activity, and higher concentrations of active PAI-1 and H3.1 nucleosomes compared to survivors (Table 3; Figure 5).

**Table 3:** Summary of coagulation, fibrinolysis and NET formation biomarkers data for dogs with sepsis that survived to hospital discharge compared to dogs that were euthanized during hospitalization.

Variable	Survivors (*n* = 22)	Non-survivors (*n* = 6)	*p*
Coagulation parameters			
Activated partial thromboplastin time (s)	14.4 (12.3–15.0)	20.5 (14.6–33.6)	**0.008**
Prothrombin time (s)	12.8 (12.2–13.3)	14.0 (12.5–16.2)	0.129
Antithrombin activity (%)	95 (83–102)	57 (40–74)	**<0.001**
Clottable fibrinogen (Clauss) (mg/dL)	1,000 (551–1,176)	535 (141–897)	0.126
D-dimer (ng/mL)	697 (293–1,311)	907 (574–4,727)	0.283
Thrombin generation			
Lag time (min)	3.0 (2.7–3.9)	3.3 (2.7–7.4)	0.720
Time to peak (min)	6.3 (5.3–7.3)	5.9 (4.9–12.7)	0.743
Peak thrombin (nM)	28.4 (20.7–34.0)	24.6 (5.6–39.2)	0.670
Endogenous thrombin potential (nM·min)	163 (138–189)	133 (57–267)	0.659
Fibrinolysis parameters			
Plasminogen (%)	114 (87–127)	51 (21–136)	0.210
Antiplasmin activity (%)	119 (115–125)	118 (81–130)	0.712
Total plasminogen activator inhibitor-1 (ng/mL)	32.5 (23.8–71.0)	69.5 (53.8–86.0)	0.209
Active plasminogen activator inhibitor-1 (ng/mL)	7.4 (0.0–10.8)	17.0 (8.9–30.6)	**0.023**
Thrombin-activatable fibrinolysis inhibitor (%)	119 (83–133)	54 (22–131)	0.090
Overall hemostatic potential (OHP) assay			
Overall coagulation potential (kOD·min)	39.52 (24.39–62.35)	29.80 (16.22–49.31)	0.331
Overall hemostatic potential (OD·min)	3,342 (1168–5,880)	10,377 (4463–13,446)	0.183
^1^ Overall fibrinolysis potential (%)	92.4 (79.3–97.1)	70.8 (59.8–87.8)	0.120
NET formation biomarkers			
Cell-free DNA (ng/mL)	495 (401–590)	633 (479–1,516)	0.100
H3.1 nucleosomes (ng/mL)	249 (77–499)	678 (401–3,003)	**0.021**

Nonparametric data are summarized as median (interquartile range) and compared between groups by Mann–Whitney U test. Corrections for multiple comparisons were not applied because these analyses were conducted based on a priori hypotheses. *P*-values in bold type are considered significant at <0.05 level.

1 Overall fibrinolysis potential (OFP) % = (OCP−OHP/OCP) * 100.

## Discussion

Dogs with sepsis have numerous potential coagulation disturbances (3, 52) and are frequently hypercoagulable (53) and hypofibrinolytic (23). The combination of hypercoagulability and hypofibrinolysis supports the hypothesis that dogs with bacterial sepsis are at risk of thrombosis (6, 7, 54, 55) due to increased propensity for clot formation combined with a reduction in clot breakdown. However, similar coagulation disturbances have been observed in dogs with other conditions (5, 56–60). Similarly, in humans, non-infectious critical illnesses are associated with coagulation and fibrinolysis disturbances comparable to those seen in sepsis (33, 35, 37). In our previous study of coagulation and fibrinolytic disorders in dogs with sepsis we used a cohort of healthy dogs as the comparator group (23). As such, the abnormalities we observed might have been common to many critical illnesses in dogs, rather than unique features of sepsis. The primary aim of this study was to address this question by comparing coagulation variables in dogs with bacterial sepsis and non-septic critical illness. Moreover, we aimed to better understand the contributions of the fibrinolysis inhibitors PAI-1 and TAFI and the NET components cfDNA and H3.1 nucleosomes to the coagulation disturbances of canine sepsis.

Two populations of dogs were enrolled, those with bacterial sepsis and those with various other injuries and illnesses that required hospitalization for intensive care. Although the inciting disorders were markedly heterogeneous, almost all the population characteristics and routine clinical and clinicopathologic variables were similar between the two groups after correction for multiple comparisons. These corrections were necessary to control type I error because large numbers of intergroup comparisons were made without being the primary focus of *a priori* hypotheses. Two differences remained significant after these corrections (MCHC and ALT), but these were not considered to be clinically relevant or of consequence for the coagulation assessments performed. Owing to our study inclusion criteria, all dogs satisfied ≥2 SIRS criteria, but most dogs in both groups satisfied 3 or more criteria, even after respiratory rate was excluded as a criterion for persistently panting dogs. The definition of sepsis (SIRS plus infection) we used for this study derives from veterinary adaptations (3, 12, 39, 61, 62) of the 2001 human medical consensus conference definition of sepsis (63). Currently, there is no consensus definition for sepsis in small animals, although efforts to derive one based on a systematic review of the literature are ongoing (64). We recognize that our use of a SIRS-based definition has limitations including excessive sensitivity (65). We likely enrolled a population of dogs that had a lower illness severity than if we had applied a sepsis definition that required identification of organ dysfunction for study eligibility. The median APPLE_full_ and APPLE_fast_ scores in our sepsis cohort here were 24 and 22, respectively. Per the mortality probability estimates from the original APPLE score derivation study (42), these medians scores equate with 7 and 18% mortality rates respectively, and it has been previously suggested that the APPLE_fast_ score might be superior for mortality risk assessment in dogs with sepsis (62). The overall mortality rate in the sepsis dogs in our study was 21% (6/28 dogs), but all these deaths resulted from euthanasia and hence our mortality rate might be higher than would have occurred naturally.

The 2016 medical sepsis 3 definition used an expected mortality figure of 10% in-hospital death as the benchmark for sepsis in humans (2), while an in-hospital mortality rate of 40% for septic shock is expected. We did not formally score or estimate the extent of organ dysfunction in our sepsis cohort aside from use of the APPLE score. However, the median values for modified Glasgow coma scale score, arterial pressure, oxygen saturation, platelet count, bilirubin and creatinine concentrations were not abnormal or outside of their respective reference intervals (see Table 1; Supplementary materials). As such, it’s likely that a minority of dogs with sepsis in this study would be classified as septic had a definition requiring >1 organ dysfunction been used. Consequently, the extent and severity of coagulation abnormalities we identified are likely less severe than in populations of septic dogs with multiple organ dysfunction. If future studies define sepsis in dogs based on identification of organ dysfunction, then our observations might not be fully replicated. Regardless, the dogs in the present study had evidence of systemic inflammation, and a life-threatening bacterial infection such that the coagulation disturbances observed provide valuable insights into the pathobiology of the sepsis syndrome.

The variety of hemostatic tests and assay endpoints in our study revealed different mechanisms of prothrombotic imbalance in dogs with sepsis, with potential for synergistic effects. High plasma fibrinogen concentration is a common finding in dogs with inflammatory disorders, because fibrinogen is a positive acute-phase protein (66–68). Dogs with sepsis had significantly higher fibrinogen than dogs with nSIRS, and their fibrinogen values were strongly correlated with fibrin clot structural properties measured as OCP. Additionally, dogs with sepsis had higher ETP values indicating their procoagulant clotting factors had a greater enzymatic potential to generate thrombin in an assay system insensitive to plasma fibrinogen content. Together, high thrombin generating capacity and high plasma fibrinogen concentration are likely to promote stable fibrin clot formation in dogs with sepsis.

The increased activities of AP and TAFI suggest that dogs with sepsis simultaneously have a fibrinolytic imbalance that could impair clot breakdown and potentiate the thrombotic risk. However, we did not detect a relative decrease in fibrinolysis as measured in the OFP assay in the sepsis group. It may be that the concentration of exogenous tPA used to initiate fibrinolysis limited the assay’s sensitivity to detect the influence of the various endogenous fibrinolysis inhibitors. We previously observed increased TAFI activity in dogs with sepsis, but did not measure total or active PAI-1 in that study (23). PAI-1 is the main physiological inhibitor of tPA and urokinase and is produced by endothelial cells, platelets and monocytes (69). Few studies of PAI-1 have been reported in dogs to date but increased PAI-1 activity was described in hyperlipidemic dogs (70), while decreased PAI-1 concentration was observed in a study of dogs with babesiosis (71). Both of those studies used different assays than employed in our study. Following our previous investigation of fibrinolysis in dogs with sepsis we speculated that increased PAI-1 concentrations might contribute to the hypofibrinolysis observed. A study of 18 dogs with sepsis from 2 centers in Australia used the same total PAI-1 assay as was used in our study (20), and identified increased PAI-1 concentrations. In those dogs, total PAI-1 concentrations ranged from 30 to 1,029 ng/mL with a median of 88 ng/mL. In our study, the PAI-1 concentrations in dogs with sepsis were generally lower (median 39 ng/mL, min-max 11–335). This may be related to illness severity because the median APPLE_fast_ score in the Australian sepsis cohort was 29 compared with 22 in our study.

In the current study, PAI-1 was not different between dogs with nSIRS and those with sepsis and no association between PAI-1 concentrations and the OFP were observed. These findings argue that although PAI-1 concentrations are higher than observed in healthy dogs, excess PAI-1 does not differentiate hemostatic imbalance among dogs with sepsis and those with nSIRS. In humans, PAI-1 is an early marker of coagulation disturbances in sepsis (72), however increased circulating PAI-1 levels also accompany chronic non-infectious inflammatory syndromes in people (73). Timing of sample collection relative to the onset of disease could not be controlled, or readily accounted for, in our study. However, it may have impacted the relative concentrations of many of the biomarkers we measured and influenced their inter-relationships.

The concentrations of the two NETosis biomarkers we measured (cfDNA and H3.1 nucleosomes) were not different between dogs with nSIRS and those with sepsis. Although we used these two variables to provide an estimate of NETosis in these dogs, they are not specific to NETs and both biomarkers may also derive from apoptotic and necrotic cells (27, 74–76). Currently, there is no readily available method to determine which cellular process leads to release of the cfDNA or H3.1 nucleosomes (27). Proposed methods for analysis of human samples include detection of citrullinated histones (77), sandwich ELISAs using antibodies to 2 distinct NET components (78), and flow cytometry (79). None of these assays are suitable for use on canine samples, however. Microscopy-based identification of NETosis is feasible in samples collected from dogs with sepsis (26), but is time-consuming, difficult to standardize and unsuitable for batch analysis.

In our study, cfDNA and H3.1 nucleosomes were strongly correlated in dogs with sepsis. This might be due to a common origin of both biomarkers, such as from NETs or that both are associated with another factor that separately increased release of these markers from distinct sources (27). Regardless the association between cfDNA concentrations and PT and the associations between H3.1 nucleosome concentrations and aPTT, D-dimers, and AT activity support the hypothesis that these cellular components participate in the immunothrombosis response initiated by sepsis (25, 80). Previous *in vitro* data suggested that canine NET components impair fibrinolysis (59), but no relationship between cfDNA or H3.1 nucleosome concentrations and OFP was observed in our study. This may be related to differences between the in vitro assay conditions and the complex heterogeneity of naturally occurring disease.

Both populations of dogs in our study had similar degrees of illness severity as quantified by the APPLE_full_ and APPLE_fast_ scores, and the mortality rates in the two populations were similar. We observed similar baseline population characteristics and clinical assessment parameters between groups and found no differences in total leukocyte, mature neutrophil and band neutrophil counts between the groups. We did not perform additional evaluations of the inflammatory status of these dogs, however. Analysis of acute phase protein or cytokine concentrations would have provided additional information about the inflammatory status of the dogs and could have enabled comparison of the degree of systemic inflammation with the coagulation disturbances. This is a limitation of our study.

The focus of our study was hemostatic imbalance associated with sepsis and thus we compared assay parameters between survivors (*n* = 26) and non-survivors (*n* = 6) within the sepsis cohort only. We found that active PAI-1 and H3.1 nucleosome concentrations were significantly higher in non-survivors and AT activity was significantly lower. Reductions in AT activity have been observed in several previous cohorts of dogs with sepsis and low AT activity has been identified as a negative prognostic indicator in multiple studies (3, 4, 52, 53, 56). High active PAI-1 and H3.1 nucleosome concentrations are novel findings and thus should be considered preliminary and hypothesis generating pending future studies including larger numbers of dogs. Consistent with our findings, a recent review of the human sepsis literature suggested that PAI-1 and NETosis biomarkers might be valuable prognostic markers (81).

Our study has limitations. We enrolled a small, heterogeneous study population including dogs with various sources of bacterial infection, species of bacteria and affected organ systems. This may have diminished the strength of any signal we observed, increasing the likelihood that abnormalities and associations were not identified. To balance patient population specificity with the need for timely completion of patient enrollment, our septic patient population included dogs for which an infection was not definitively documented. Dogs were eligible for enrollment if they had a high index of suspicion for infection, consistent with other investigators in the field (40, 41, 82), but this could have led to misclassification of dogs as having sepsis rather than nSIRS. The clinical caseload precluded standardizing the diagnostic testing performed for each dog due to client cost constraints. Bacterial cultures were lacking for several dogs in the sepsis group; a potential cause of misclassification. Before analyzing stored samples, we thoroughly reviewed the medical records for all enrolled dogs to confirm correct group assignment. This review included all available diagnostic test results, clinical response to treatment and data from subsequent re-evaluations. We made every effort to correctly assign dogs to their respective group but acknowledge that we did not definitively document infection in some dogs assigned to the sepsis group or eliminate the possibility of occult infections in dogs in the nSIRS group.

We used prior data on TAFI activity to estimate the required sample size, but those data were for comparisons of dogs with sepsis and healthy controls. Variability in coagulation parameters is likely to be a function of the populations studied. Our sample size may have been insufficient to detect differences in other analytes such as PAI-1. The overall illness severity in our study was moderate and the timing of sampling relative to disease onset was variable. These factors likely influenced the concentrations of the measured parameters and the relationships and differences we observed. The number of non-survivors in this cohort was small and hence our findings with respect to outcome should be considered exploratory. The limitations of clinical observational studies enrolling client-owned dogs presenting to specialty hospitals include non-uniform treatment prior to presentation and sample acquisition. The type and extent of treatment administered before study enrollment was likely influenced by illness severity further complicating evaluation of the relationships between biomarkers and illness severity scores. Experimental induction of sepsis would eliminate these confounding influences because the type, timing, location, and nature of the septic focus can be controlled, and samples obtain serially throughout the course of disease progression, treatment and recovery. The obvious ethical dilemmas associated with such large animal models of sepsis might preclude obtaining those data, however. Moreover, homogenous, tightly controlled experimental models of sepsis lack a general applicability to real-world situations involving naturally occurring disease. Ideally, large multicenter observational studies, with uniform enrollment and stratification criteria, could help overcome some of the limitations of our single-center investigation.

In summary, in this single center study of dogs with sepsis and non-septic critical illness, dogs with sepsis were hyperfibrinogenemic, hypercoagulable and had higher AP and TAFI activities relative to critically ill dogs without infection. This supports the use of coagulation assays to monitor for complications in dogs with sepsis and the targeted use of thromboprophylaxis in patients with disturbances that might predispose them to clinically relevant thrombosis. Concentrations of H3.1 nucleosomes and active PAI-1 and AT activity might have prognostic value in dogs with sepsis and these markers warrant further investigation in other cohorts of dogs.

## Data Availability

The raw data supporting the conclusions of this article will be made available by the authors, without undue reservation.

## Supplementary material

SUPPLEMENTARY FIGURE S1 Dotplots of **(A)** activated partial thromboplastin time (aPTT, s), **(B)** prothrombin time (PT, s), **(C)** antithrombin activity (%), and **(D)** D-dimer concentration (ng/mL) in dogs with sepsis compared to nSIRS. Data distribution determined whether an unpaired *t*-test with Welch’s correction (AT activity only) or the Mann-Whitney U test were used for group comparisons.

SUPPLEMENTARY FIGURE S2 Dotplots representing derived thrombin generation parameters where **(A)** represents the lag time, **(B)** the time to reach peak thrombin formation, and **(C)** peak thrombin concentration of dogs with sepsis compared to dogs with nSIRS. Data were compared with the Mann-Whitney U test, with alpha set at 0.05.

SUPPLEMENTARY FIGURE S3 Dotplots of **(A)** overall fibrinolysis potential (OFP), **(B)** plasminogen activity (%), **(C)** total plasminogen activator inhibitor-1 (PAI-1), and **(D)** active PAI-1 concentrations. Data were compared with the Mann-Whitney U test, with alpha set at 0.05.

SUPPLEMENTARY FIGURE S4 Dotplots of **(A)** cell-free DNA (ng/mL) and **(B)** H3.1 nucleosomes (ng/mL) in dogs with bacterial sepsis compared to nSIRS. Data were compared with the Mann-Whitney U test, with alpha set at 0.05.

SUPPLEMENTARY TABLE S1 Summary data and between group comparisons of point-of-care venous blood gas, electrolyte and metabolite analyses.

SUPPLEMENTARY TABLE S2 Summary data and between group comparisons of complete blood count analyses.

SUPPLEMENTARY TABLE S3 Summary data and between group comparisons of serum biochemistry panels.

SUPPLEMENTARY TABLE S4 Bacterial pathogens cultured from dogs diagnosed with bacterial sepsis.
