# Supplementary figures and images for: Dogs with sepsis are more hypercoagulable and have higher fibrinolysis inhibitor activities than dogs with non-septic systemic inflammation
Source: Front Vet Sci. 2025 Apr 30;12:1559994. doi: 10.3389/fvets.2025.1559994 (PMC12075940; doi:10.3389/fvets.2025.1559994)

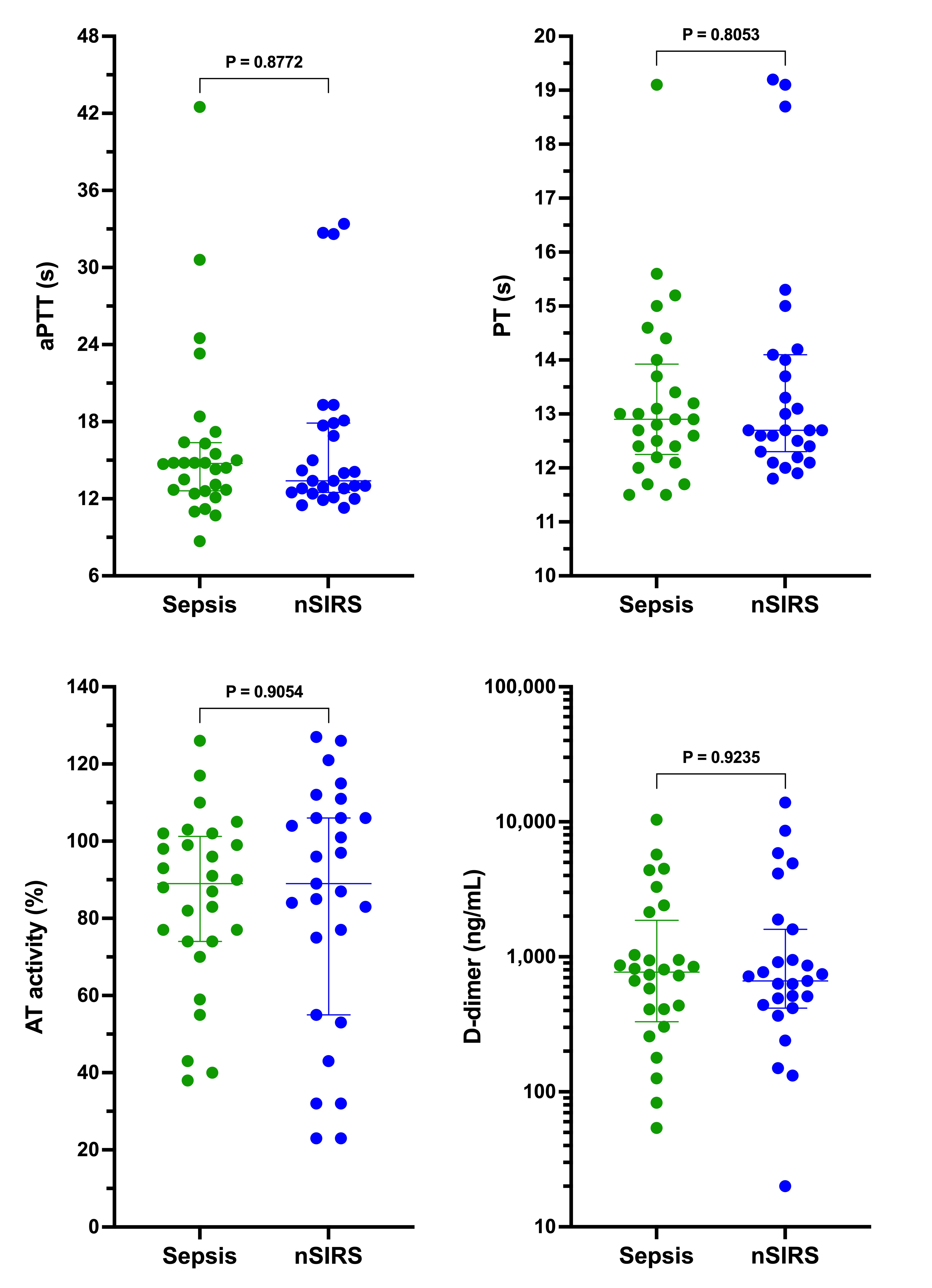

Supplement: SUPPLEMENTARY FIGURE S1 — Dotplots of (A) activated partial thromboplastin time (aPTT, s), (B) prothrombin time (PT, s), (C) antithrombin activity (%), and (D) D-dimer concentration (ng/mL) in dogs with sepsis compared to nSIRS. Data distribution determined whether an unpaired t-test with Welch’s correction (AT activity only) or the Mann-Whitney U test were used for group comparisons. [file Image_1.JPEG]

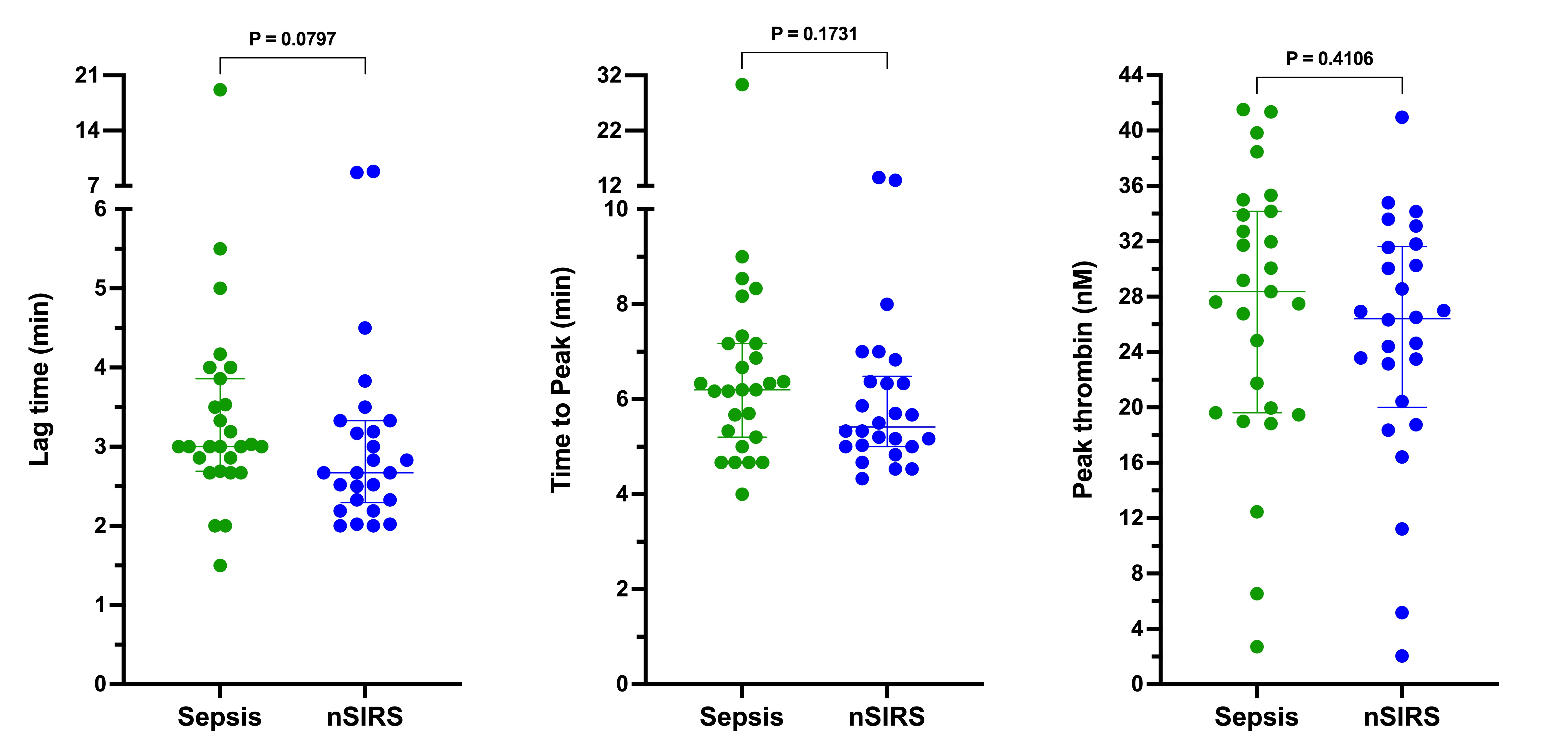

Supplement: SUPPLEMENTARY FIGURE S2 — Dotplots representing derived thrombin generation parameters where (A) represents the lag time, (B) the time to reach peak thrombin formation, and (C) peak thrombin concentration of dogs with sepsis compared to dogs with nSIRS. Data were compared with the Mann-Whitney U test, with alpha set at 0.05. [file Image_2.JPEG]

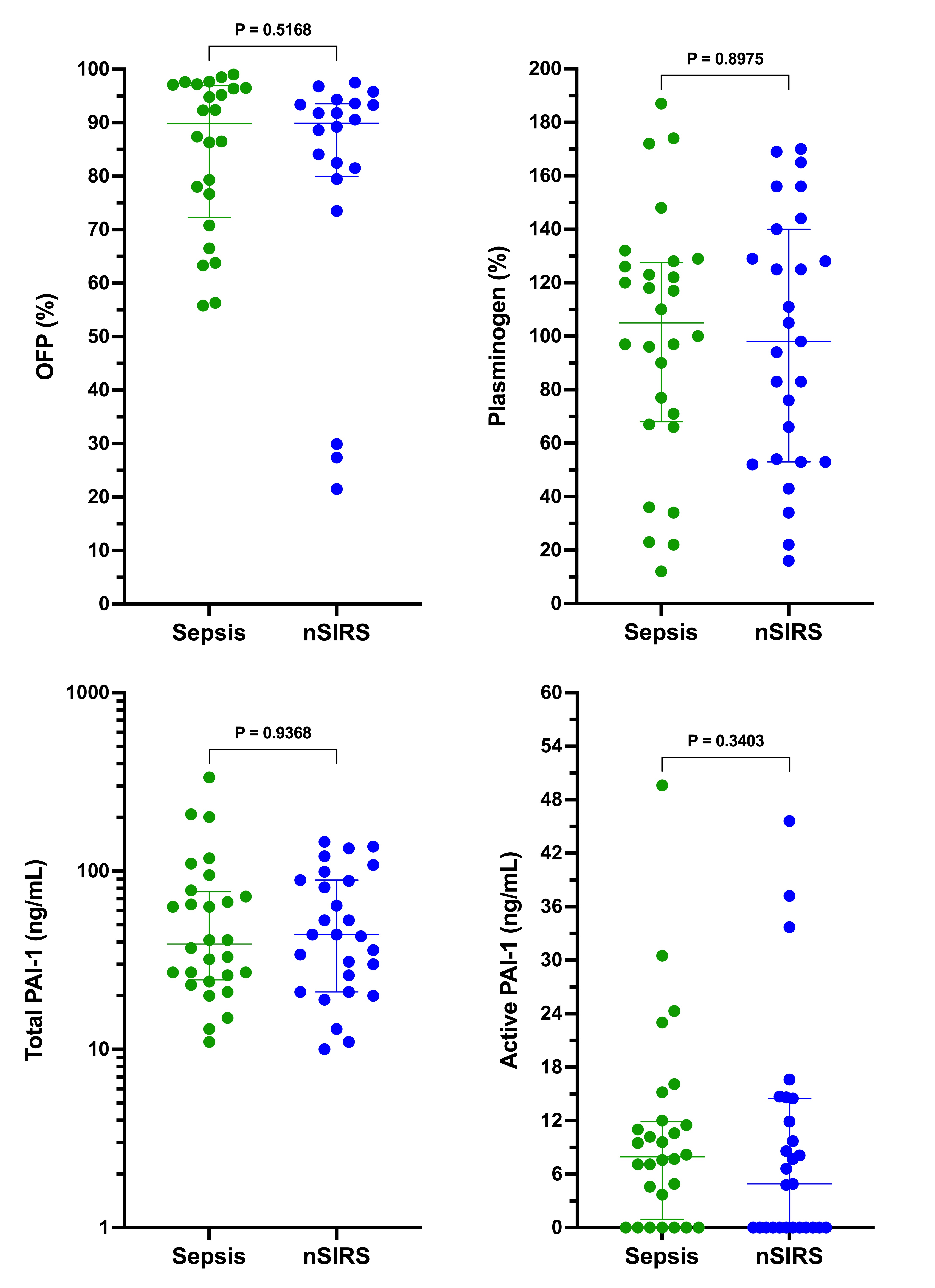

Supplement: SUPPLEMENTARY FIGURE S3 — Dotplots of (A) overall fibrinolysis potential (OFP), (B) plasminogen activity (%), (C) total plasminogen activator inhibitor-1 (PAI-1), and (D) active PAI-1 concentrations. Data were compared with the Mann-Whitney U test, with alpha set at 0.05. [file Image_3.JPEG]

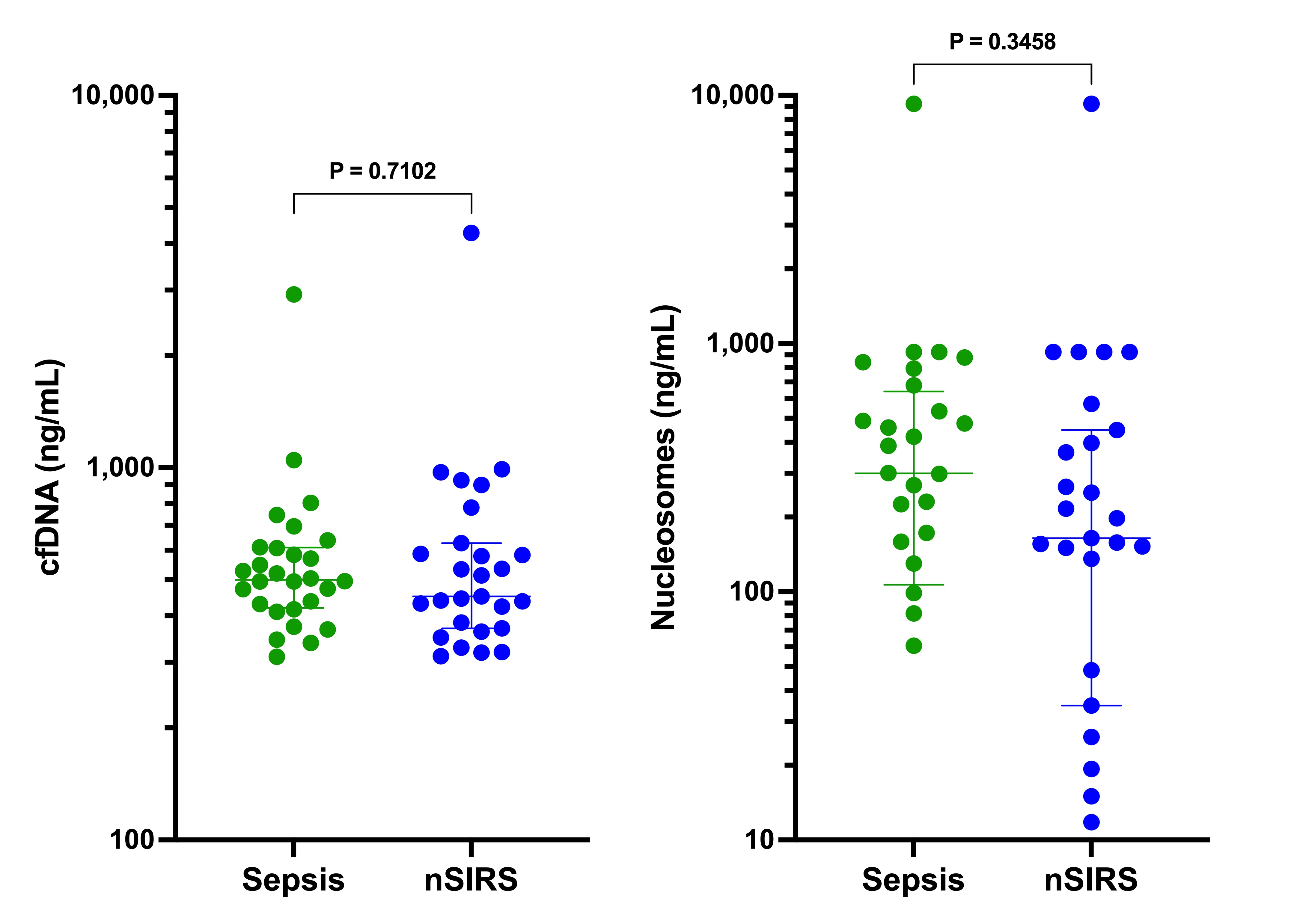

Supplement: SUPPLEMENTARY FIGURE S4 — Dotplots of (A) cell-free DNA (ng/mL) and (B) H3.1 nucleosomes (ng/mL) in dogs with bacterial sepsis compared to nSIRS. Data were compared with the Mann-Whitney U test, with alpha set at 0.05. [file Image_4.JPEG]
