# Supplementary material for: Dogs with sepsis are more hypercoagulable and have higher fibrinolysis inhibitor activities than dogs with non-septic systemic inflammation
Source: Front Vet Sci. 2025 Apr 30;12:1559994. doi: 10.3389/fvets.2025.1559994 (PMC12075940; doi:10.3389/fvets.2025.1559994)
Supplement: SUPPLEMENTARY TABLE S1 — Summary data and between group comparisons of point-of-care venous blood gas, electrolyte and metabolite analyses. [file Table_1.DOCX]

| **Variable (unit)** | **Reference interval** | **Sepsis** | **nSIRS** | **Unadjusted P** |
| --- | --- | --- | --- | --- |
| pH | 7.32-7.38 | 7.38 ± 0.07 | 7.35 ± 0.08 | .243 |
| p_v_CO_2_ (mmHg) | 38-46 | 33.7 (29.2-38.5) | 36.6 (29.9-42.6) | .264 |
| HCO_3_^-^ (mmol/L) | 20-25 | 19.9 ± 3.4 | 19.6 ± 4.0 | .776 |
| BE (mmol/L) | -4 to 0 | -5.2 ± 3.9 | -6.0 ± 4.6 | .551 |
| Na^+^ (mmol/L) | 145-151 | 144 (142-147) | 147 (143-150) | .030* |
| K^+^ (mmol/L) | 3.9-5.1 | 4.0 (3.9-4.2) | 4.1 (3.8-4.4) | .772 |
| Cl^-^ (mmol/l) | 110-119 | 109 (105-112) | 110 (108-112) | .442 |
| iCa^2+^ (mmol/L) | 1.18-1.37 | 1.28 (1.24-1.32) | 1.27 (1.23-1.31) | .709 |
| Glucose (mg/dL) | 60-120 | 101 (87-109) | 110 (99-128) | .036* |
| Hematocrit (%) | 42-57 | 48 (42-57) | 46 (40-50) | .326 |
| Lactate (mmol/L) | 0.0-2.0 | 2.3 (1.5-3.0) | 2.6 (1.4-5.8) | .509 |

*Bonferroni corrected P-values (n=11 comparisons): Na^+^ = .327; Glucose = .398.
