# Supplementary material for: Dogs with sepsis are more hypercoagulable and have higher fibrinolysis inhibitor activities than dogs with non-septic systemic inflammation
Source: Front Vet Sci. 2025 Apr 30;12:1559994. doi: 10.3389/fvets.2025.1559994 (PMC12075940; doi:10.3389/fvets.2025.1559994)
Supplement: SUPPLEMENTARY TABLE S2 — Summary data and between group comparisons of complete blood count analyses. [file Table_2.DOCX]

| **Variable (unit)** | **Reference interval** | **Sepsis** | **nSIRS** | **Unadjusted P** |
| --- | --- | --- | --- | --- |
| Hematocrit (%) | 41-58 | 46.2 ± 9.8 | 45.7 ± 9.3 | .851 |
| Hemoglobin (g/dL) | 14.1-20.1 | 15.4 ± 3.0 | 14.9 ± 3.2 | .552 |
| Erythrocytes (×10^3^/µL) | 5.7-8.5 | 6.3 ± 1.2 | 6.2 ± 1.3 | .744 |
| MCV (fL) | 64-76 | 73 (71-76) | 73 (71-76) | .818 |
| MCH (pg) | 21-26 | 24.4 ± 1.2 | 24.0 ± 1.2 | .217 |
| MCHC (g/dL) | 33-36 | 33.0 (33.0-34.0) | 32.5 (32.0-33.0) | **<0.001*** |
| RDW (%) | 10.6-14.3 | 12.4 (12.0-12.7) | 12.4 (12.0-12.8) | .898 |
| Reticulocytes (%) | 0.2-1.5 | 0.5 (0.3-1.1) | 0.8 (0.6-1.4) | .023* |
| Absolute reticulocytes (×10^3^/µL) | 11-92 | 35 (16-71) | 49 (37-88) | .057 |
| Nucleated RBCs / 100 WBC | 0-1 | 0 (0-0) | 0 (0-1) | .170 |
| Total leukocytes (×10^3^/µL) | 5.7-14.2 | 17.5 ± 9.9 | 15.1 ± 5.2 | .274 |
| Neutrophils (×10^3^/µL) | 2.7-9.4 | 13.6 ± 8.7 | 11.5 ± 4.0 | .253 |
| Band neutrophils (×10^3^/µL) | 0.0-0.1 | 0.7 (0.2-1.9) | 0.2 (0.0-0.8) | .095 |
| Band neutrophils (%) | - | 4.5 (1.0-13.5) | 1.1 (0.0-5.9) | .041* |
| Lymphocytes (×10^3^/µL) | 0.9-4.7 | 1.1 ± 0.7 | 1.4 ± 0.8 | .251 |
| Monocytes (×10^3^/µL) | 0.1-1.3 | 1.0 (0.4-1.8) | 0.7 (0.4-1.1) | .344 |
| Eosinophils (×10^3^/µL) | 0.1-2.1 | 0.0 (0.0-0.2) | 0.2 (0.0-0.5) | .072 |
| Basophils (×10^3^/µL) | 0.0-0.1 | 0.0 (0.0-0.0) | 0.0 (0.0-0.0) | .317 |
| Platelets (×10^3^/µL) | 186-545 | 213 ± 140 | 204 ± 109 | .784 |
| MPV (fL) | 8.4-14.1 | 12.1 (10.2-13.5) | 11.5 (9.8-12.6) | .178 |

*Bonferroni corrected P-values (n=20 comparisons): MCHC = .013; Reticulocytes = .466; Band neutrophil (%) = .820.
