# Supplementary material for: Dogs with sepsis are more hypercoagulable and have higher fibrinolysis inhibitor activities than dogs with non-septic systemic inflammation
Source: Front Vet Sci. 2025 Apr 30;12:1559994. doi: 10.3389/fvets.2025.1559994 (PMC12075940; doi:10.3389/fvets.2025.1559994)
Supplement: SUPPLEMENTARY TABLE S3 — Summary data and between group comparisons of serum biochemistry panels. [file Table_3.DOCX]

| **Variable (unit)** | **Reference interval** | **Sepsis** | **nSIRS** | **Unadjusted P** |
| --- | --- | --- | --- | --- |
| Sodium (mEq/L) | 143-150 | 144 ± 4 | 146 ± 5 | .038* |
| Potassium (mEq/L) | 4.1-5.4 | 4.1 (3.8-4.5) | 4.0 (3.8-4.3) | .481 |
| Chloride (mEq/L) | 106-114 | 108 (104-112) | 111 (107-112) | .153 |
| Bicarbonate (mEq/L) | 14-24 | 17.4 ± 3.4 | 18.3 ± 3.7 | .330 |
| Anion gap (mEq/L) | 17-27 | 22.5 (20.0-24.0) | 21.0 (20.0-25.0) | .623 |
| Blood urea nitrogen (mg/dL) | 9-26 | 14.5 (10.0-20.0) | 16.0 (12.0-20.0) | .728 |
| Creatinine (mg/dL) | 0.6-1.4 | 0.9 (0.7-1.2) | 0.9 (0.8-1.3) | .468 |
| Total calcium (mg/dL) | 9.4-11.1 | 9.6 ± 0.8 | 9.5 ± 0.9 | .699 |
| Inorganic phosphate (mg/dL) | 2.7-5.4 | 4.4 (3.9-5.1) | 4.0 (3.3-4.7) | .213 |
| Total magnesium (mEq/L) | 1.5-2.1 | 1.7 (1.4-1.9) | 1.7 (1.6-2.0) | .275 |
| Total protein (g/dL) | 5.5-7.2 | 6.0 ± 1.7 | 5.6 ± 1.2 | .376 |
| Albumin (g/dL) | 3.2-4.1 | 2.7 (2.1-3.4) | 3.4 (2.3-3.9) | .084 |
| Globulin (g/dL) | 1.9-3.7 | 2.8 (2.2-3.7) | 2.5 (2.0-2.8) | .111 |
| Glucose (mg/dL) | 68-104 | 96 (77-105) | 103 (85-110) | .203 |
| Alanine aminotransferase (U/L) | 17-95 | 49 (31-54) | 152 (62-294) | **<.001*** |
| Aspartate transaminase (U/L) | 18-56 | 59 (42-186) | 151 (88-213) | .014* |
| Alkaline phosphatase (U/L) | 7-115 | 141 (111-204) | 127 (74-367) | .637 |
| Ɣ-glutamyl transferase (U/L) | 0.0-8.0 | 2.0 (0.8-3.5) | 2.0 (2.0-5.0) | .255 |
| Total bilirubin (mg/dL) | 0.0-0.2 | 0.1 (0.1-0.2) | 0.1 (0.1-0.5) | .147 |
| Direct bilirubin (mg/dL) | 0.0-0.1 | 0.1 (0.0-0.1) | 0.1 (0.0-0.2) | .294 |
| Indirect bilirubin (mg/dL) | 0.0-0.1 | 0.1 (0.0-0.1) | 0.1 (0.1-0.2) | .265 |
| Amylase (U/L) | 322-1310 | 722 (413-990) | 668 (572-1029) | .600 |
| Lipase (U/l) | 15-228 | 55 (20-121) | 97 (41-458) | .040* |
| Cholesterol (mg/dL) | 136-392 | 254 ± 94 | 199 ± 98 | .042* |
| Creatine kinase (U/L) | 64-314 | 345 (169-1047) | 812 (310-2105) | .056 |
| Lactate dehydrogenase (U/L) | 24-388 | 222 (108-380) | 162 (93-197) | .220 |
| Total iron (µg/dL) | 97-263 | 47 (32-81) | 91 (53-178) | .012* |
| Total iron binding capacity (µg/dL) | 280-489 | 294 ± 95 | 362 ± 81 | .007* |
| Iron saturation (%) | 27-66 | 24 ± 15 | 37 ± 28 | .047* |

*Bonferroni corrected P-values (n=29 comparisons): Sodium = 1.00; ALT = .007; AST = .418; Lipase = 1.00; Cholesterol = 1.00; Total iron = .333; Total iron binding capacity = .212; Iron saturation 1.00.
