# Supplementary material for: Dogs with sepsis are more hypercoagulable and have higher fibrinolysis inhibitor activities than dogs with non-septic systemic inflammation
Source: Front Vet Sci. 2025 Apr 30;12:1559994. doi: 10.3389/fvets.2025.1559994 (PMC12075940; doi:10.3389/fvets.2025.1559994)
Supplement: SUPPLEMENTARY TABLE S4 — Bacterial pathogens cultured from dogs diagnosed with bacterial sepsis. [file Table_4.DOCX]

Table S4. Bacterial pathogens cultured from dogs diagnosed with bacterial sepsis.

| **Bacterial pathogen** | **Dogs with sepsis (n=28)** |
| --- | --- |
| *Escherichia coli* | 5 |
| *Klebsiella pneumoniae* | 2 |
| *Fusobacterium* spp. | 3 |
| *Clostridium* spp. | 2 |
| *Proteus mirabilis* | 1 |
| *Enterococcus* spp. | 3 |
| *Terrisporobacter glycolicus* | 1 |
